# Epidemiological, Clinical, and Molecular Insights into Canine Distemper Virus in the Mekong Delta Region of Vietnam

**DOI:** 10.3390/v17060781

**Published:** 2025-05-29

**Authors:** Tien My Van, Dao Thi Anh Tran, Chien Tran Phuoc Nguyen, Giang Truong Huynh, Mong Thi Nhu Luu, Trung Quang Le, Bich Ngoc Tran

**Affiliations:** 1 Faculty of Veterinary Medicine, Can Tho University, Can Tho City 94000, Vietnam; vanmytien@gmail.com (T.M.V.); daotta@vlute.edu.vn (D.T.A.T.); ntpchien@ctu.edu.vn (C.T.P.N.); htgiangty@ctu.edu.vn (G.T.H.); ltnmong@ctu.edu.vn (M.T.N.L.); lqtrung@ctu.edu.vn (T.Q.L.); 2 Faculty of Applied Biological Sciences, Vinh Long University of Technology and Education, Vinh Long 85000, Vietnam

**Keywords:** canine distemper, dog, epidemiological characterization, F gene, genetic analyses, H gene, Mekong Delta

## Abstract

Canine distemper virus (CDV) is a highly contagious pathogen and causes a fatal systemic disease in domestic dogs and wild carnivores worldwide. Despite CDV infections being monitored globally, studies on CDV in Vietnam seem to be limited. This study, therefore, investigated the epidemiological, clinical, and molecular characteristics of CDV in the Mekong Delta (MD) region of Vietnam. A total of 6687 ocular/nasal swabs were collected from CDV-suspected dogs across seven cities/provinces. CDV infection was detected in 6.19% (414 dogs) of suspected dogs using a commercially available rapid kit, with infection associated with age, roaming status, and vaccination status. Hematological and blood biochemical analysis of CDV-infected dogs revealed anemia, leukopenia, neutrophilia, thrombocytopenia, a slight increase in aspartate aminotransferase (AST) levels, and a significant increase in blood urea nitrogen (BUN) levels. Molecular characterization of partial hemagglutinin (H) and fusion (F) genes exhibited high nucleotide and amino acid homology with the Asia-1 genotype. Phylogenetic analysis confirmed that the field sequences were clustered into the Asia-1 genotype together with the neighboring countries. These findings provide important insights into the current epidemiological, clinical, and molecular features of CDV circulating in Vietnam.

## 1. Introduction

Canine distemper (CD) is a highly transmissible infectious and fatal systemic disease in domestic dog populations and wild carnivores globally, caused by the canine distemper virus (CDV) which is a single-stranded RNA virus belonging to the *Morbillivirus* genus and *Paramyxoviridae* family [1,2]. CDV complete viral genome contains 15,690 nucleotides, encodes six structural proteins including hemagglutinin (H), fusion (F), large (L), matrix (M), phospho (P), termed nucleocapsid (N), and two non-structural proteins (C and V). The H and F glycoproteins play a substantial role in the adsorption and fusion of the virus to the host cells. Both H and F glycoproteins are the epitopes and induce the immune response against pathogens [2,3]. The H gene of CDV contains 1824 nucleotides in length and encodes 607 amino acids. There are approximately eight to ten potential N-linked glycosylation sites (19–21, 149–151, 309–311, 339–341, 391–393, 422–424, 456–458, 542–544, 584–586, 587–589, 603–605) that are highly conserved in the H protein of CDV [4,5,6]. Meanwhile, the F gene contains 1989 nucleotides in length, encodes 662 amino acids, and comprises three regions, including Fsp, F2, and F1. There are three potential N-linked glycosylation sites (141–143, 173–175, and 179–181) in the F2 region and one potential N-linked glycosylation sites (517–519) in the F1 region [7,8,9]. Currently, the H gene is used to classify the genetic diversity of CDV among geographical regions due to its genetic variability. According to the classification of the H gene, CDV is separated into several important genotype variants, including Africa-1, Africa-2, America-1, America-2, America-3, America-4, Asia-1, Asia-2, Asia-3, Asia-4, Asia-5, Asia-6, Europe/South American-1, Europe-2/European wildlife, Europa-3/Arctic-like, South America-2, South America-3, and new clusters distributing in several geographical regions worldwide [10]. Most vaccine strains of CDV were classified into the America-1 genotype, except for the Rockborn-like vaccine strain [11]. In addition, the F gene was additionally noted as a target gene to classify genetic strains of CDV in the same way as the H gene in the last decades [8,12,13]. Therefore, the H and F genes have a prominent role in the molecular investigations of CDV [13,14], and studies on the molecular features of CDV in dog populations globally based on the H and F genes should be continuously promoted.

At present, CD is still a transboundary viral disease of domestic dog populations and wildlife worldwide, and various studies on the epidemiological and molecular characteristics of the new CDV variants have been reported in many geographical regions [7,8,9,12,13,14,15,16,17,18]. However, studies on CDV circulating in dog populations seem limited in Vietnam since the first published information on the field CDV strains in the North region in 2009, especially studies reporting on the epidemiological, clinical, and molecular characteristics of the field CDV strains in the Mekong Delta (MD) region of Vietnam [19,20,21,22]. Notably, several genotypes of CDV were found in Vietnam, including America-1, Asia-1, and/or novel clades of the Asia-1 genotype based on the H and/or P genes [19,20,21,22]. Nevertheless, there has been no previous report on the molecular characterization of the field CDV sequences isolated in dogs in Vietnam based on the partial F genes. The current study therefore first documented epidemiological, clinical, and molecular findings of CDV obtained from clinically suspected dogs in seven provinces/cities in the MD region of Vietnam based on the partial H and F genes. The results of this study highlight some basic information on the epidemiological, clinical, and molecular features of CDV circulating in dogs in the MD region and supplement additional data on CDV circulating in Vietnam.

## 2. Materials and Methods

### 2.1. Sampling

**Figure 1:**
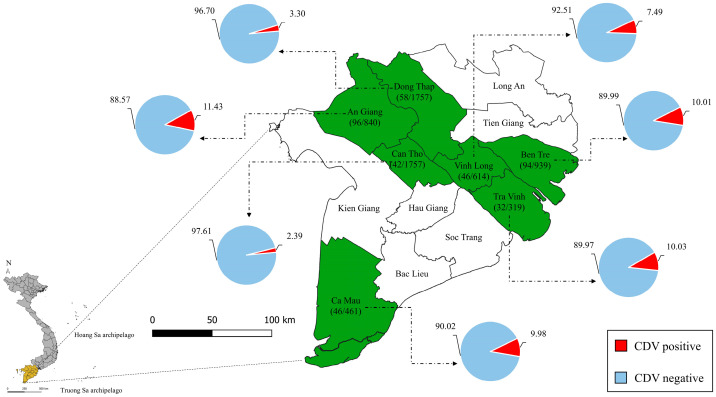
Distribution of CDV sampling in MD region of Vietnam. Selected provinces/cities for CDV sampling are highlighted in green, and numbers in parentheses indicate positive samples via CDV rapid kit/total investigated dogs. Pie charts indicate CDV-positive frequency (red part) in dogs in each province/city.

A cross-sectional study was performed over the period from 2022 to 2023 to examine 6687 dogs admitted for the first time to the veterinary clinics in An Giang Province (840 dogs), Ben Tre Province (939 dogs), Ca Mau Province (461 dogs), Can Tho City (1757 dogs), Dong Thap Province (1757 dogs), Tra Vinh Province (319 dogs), and Vinh Long Province (614 dogs) in the MD region of Vietnam (Figure 1). All admitted dogs were first screened for CDV-suspected clinical signs by veterinarians, and only dogs with the clinical signs of CDV were moved to the next step. For detection of CDV via clinical diagnostic, dogs with clinical signs such as fever, mucus ocular/nasal discharge, conjunctivitis, tachypnoea, coughing, vomiting, loss of appetite, diarrhea, bloody diarrhea, dehydration, foot pad and nasal hyperkeratosis, and nervous signs were investigated. Subsequent to the clinical diagnosis, all CDV-suspected dogs were moved to the next step, where ocular/nasal swabs were collected and tested individually via a commercially available rapid kit (Product No. RG1103DD, Gyeonggi-do, Republic of Korea). Following the test procedure of the rapid kit, 414 CDV-positive dogs were identified via a rapid kit (Product No. RG1103DD, Gyeonggi-do, Republic of Korea). Ocular/nasal swabs were then collected from 414 CDV-positive dogs with clinical signs of CDV infection as a routine diagnostic process in veterinary clinics. The samples were then stored in an Eppendorf tube containing 0.50 mL of phosphate-buffered saline (pH = 7.4) at 2–8 °C until used for further processing. Moreover, approximately 2 mL of total blood from 414 CDV-positive dogs were collected to analyze the hematology and blood biochemistry as a routine diagnostic process in veterinary clinics. Information about the purpose of the sample collection was fully provided to the owners, and informed consent was obtained prior to the sampling. All the necessary information about the investigated dogs, such as age, sex, breed, roaming status, vaccination status, and clinical signs, were carefully documented in the medical records for the individual dogs.

### 2.2. Hematology and Blood Biochemistry

Hematology and blood biochemistry tests were performed in the Practical Veterinary Clinic of Can Tho University, Can Tho City, Vietnam. All the blood samples collected from dogs in other veterinary clinics of other provinces were transported to the Practical Veterinary Clinic of Can Tho University within 24 h under cool temperature conditions at 2–8 °C. Approximately 2 mL of total blood from 414 CDV-positive dogs were collected and divided into two equal parts, one for hematology (EDTA tube) and one for blood biochemistry (Heparin tube). Blood samples were tested for hematological parameters including red blood cells (RBC), hematocrit (HCT), mean corpuscular volume (MCV), hemoglobin (HGB), mean corpuscular hemoglobin (MCH), mean corpuscular hemoglobin concentration (MCHC), white blood cell (WBC), neutrophil, lymphocyte, monocyte, eosinophil, basophil, and platelet (PLT). In addition, aspartate transaminase (AST), alanine transaminase (ALT), blood urea nitrogen (BUN), and creatinine were carried out as the blood biochemistry parameters.

### 2.3. RNA Extraction, Reverse Transcriptase Polymerase Chain Reaction (RT-PCR), and Sequencing of the Partial H and F Gene of CDV

Total viral RNA extraction and cDNA synthesis following the protocol of the previous publication [22], using the SV Total RNA Isolation kit (Promega, Madison, WI, USA) and GoScript™ (Promega, Madison, WI, USA) reverse transcription kit, respectively.

Partial H and F genes of the CDV samples collected from dogs in the MD region were amplified using specific primer pairs with 613 bp and 594 bp in length, respectively. cDNA synthesized from the ocular/nasal swabs was used as a template. For amplification, previously published primer pairs for H and F genes were utilized in the present study [8,23], the forward and reverse primers of the H gene (613 bp) were as follows: Forward: 5′-TGGTTCACAAGATGGTATTC-3′ and Reverse: 5′-CAACACCACTAAATTGGACT-3′, and the forward and reverse primers of the F gene (594 bp) were as follows: Forward: 5′-CGAGATCTAGGGTCCAGGACATAGCAAGC-3′ and Reverse: 5′-AGTTTTATGACCAAGTAC-3′. The reaction was prepared in a total volume of 25 µL including 10 µL Go Taq^®^ Green Master Mix 2X (Promega, Madison, WI, USA), 1 µL MgCl_2_ (25 mM), 0.50 µL M-MLV, 0.50 µL forward primer, 0.50 µL reverse primer, 4 µL extracted RNA sample, and 8.50 µL ultrapure distilled water free of DNase and RNase enzymes. The RT-PCR cycling conditions followed the prior studies [8,23]. The amplification products were visualized by 1.50% agarose gel electrophoresis in Tris-Borate-EDTA (TBE). The productions subsequent to purification were then sent to the commercial company in Vietnam for sequencing, using a Sanger method.

In this study, only forty-five samples were collected from forty-five CDV-positive dogs via a rapid kit (Product No. RG1103DD, Gyeonggi-do, Republic of Korea) and RT-PCR, and showed clinical signs of CDV infection that were chosen for sequencing the H and F genes via the Sanger method in the commercial company due to the limit of this study. Samples were selected based on the typical characteristics of CDV-positive dogs, including age, sex, breed, roaming status, vaccination status, and clinical signs, to reflect a range of epidemiologically relevant variables, allowing for reliable molecular features analysis. Therefore, a total of 8 CDV-positive samples from An Giang Province, 5 samples from Ben Tre Province, 5 samples from Ca Mau Province, 10 samples from Can Tho City, 5 samples from Dong Thap Province, 6 samples from Tra Vinh Province, and 6 samples from Vinh Long Province were chosen for sequencing. Detailed information about the chosen samples including GenBank accession number of the H and F gene, collected year, age, sex, vaccination status, and clinical signs of CDV-positive dogs were mentioned in Table S1.

### 2.4. Phylogenetic, Recombination, and Potential N-Linked Glycosylation Sites Analysis

The obtained sequences of the partial H and F genes selected from forty-five dogs in the MD region were initially compared to the identities/variations of reference sequences of the circulating CDV strains worldwide in the GenBank (NCBI) database using the BLASTn tool (https://blast.ncbi.nlm.nih.gov/Blast.cgi) (accessed on 9 April 2025). Following, the alignment of nucleotide sequences was conducted by the BioEdit software package version 7.2.5 [24] using the Clustal-W method. The homology of nucleotide and deduced amino acid sequences of the partial H and F genes were analyzed via the BioEdit software package version 7.2.5. Furthermore, the phylogenetic trees of CDV based on the partial H and F genes were constructed by using the maximum likelihood method of the MEGA software package version 7.0.26 with 1000 bootstrap replicates and Tamura-3 model with gamma distribution (T92 + G) model [25], and were displayed via iTOL software package version 7.1 [26]. The reference sequences of the H and F genes were harnessed from the GenBank database based on a previous study [14] and were documented in Table S2.

In addition, all CDV sequences obtained in the MD region of Vietnam were screened for potential genetic recombination by the Recombination Detection Program (RDP4) software package version 4.0, including seven integrated algorithms: RDP, GeneConv, Chimera, MaxChi, SiScan, 3Seq, and BootScan [27]. To guarantee the reliability and consistency of the findings, the obtained CDV sequences were only classified as potential genetic recombinants when at least four of the seven analytical methods produced statistically significant results, with *p*-values less than 0.01. Recombination was assessed using the putative recombinant as a query against their parent lineages, which were inferred through the RDP algorithm. Potentially recombinant sequences subsequent to calculation were visualized via the SimPlot software package version 3.5.1 [28].

Moreover, the NetNGlyc software package version 1.0 [29] was utilized to analyze the potential N-linked glycosylation sites of the field CDV sequences.

### 2.5. Data Analysis

Data collected from the survey were calculated using Microsoft Excel software package version 2016. Statistical analyses were performed using the Chi-square test on Minitab software package version 16.0, with statistical significance at *p* lower than 0.05. The WinEpi software package (http://www.winepi.net/) (accessed on 19 March 2025) was used to calculate the odds ratio (OR). The heatmap was drawn by the TBtools-II software package version 1.0 [30]. The QGIS software package version 3.22.6 was used to construct the map.

## 3. Results

### 3.1. Epidemiological Features

Subsequently to the survey, data were analyzed and the results indicated that the infection rate of CDV in dogs raised in some provinces/cities in the MD region accounted for 6.19% (414/6687 dogs) of the total number of investigated dogs via clinical diagnostic and rapid kit. The infection rate was high in An Giang Province (11.43%, 96/840 dogs), Tra Vinh Province (10.03%, 32/319 dogs), Ben Tre Province (10.01%, 94/939 dogs), and Ca Mau Province (9.98%, 46/461 dogs); however, it was low in Vinh Long Province (7.49%, 46/614 dogs), Dong Thap Province (3.30%, 58/1757 dogs), and Can Tho City (2.39%, 42/1757 dogs) (Figure 1).

According to the results, the infection rate of puppies (<6 months old) (11.68%) and old dogs (>5 years) (7.80%) was significantly higher compared to other age groups (*p* < 0.01). There was no significant difference in the infection rate between pure/cross (6.66%) and local dog breeds (5.84%) (*p* > 0.05) or between male (6.56%) and female (5.80%) dogs (*p* > 0.05). However, the infection rate of free-roaming dogs (7.28%) was significantly higher than that of confined dogs (4.81%) (*p* < 0.01). The highest rate of CDV infection was observed in unvaccinated dogs (13.67%), followed by one-dose vaccinated dogs (4.55%); meanwhile, the lowest infection rate was found in two-dose vaccinated dogs (0.87%) (*p* < 0.01). Moreover, based on the OR analysis, age, roaming status, and vaccination status of the illness dogs were found to be risk factors for CDV. However, breed and sex were not associated with the prevalence of CD in dogs in the MD (Table 1).

### 3.2. Clinical Signs

CDV-positive dogs exhibited typical clinical signs of CDV infection such as fever, lethargy, loss of appetite, vomiting, and dehydration (97.58%); ocular discharge and conjunctivitis (87.44%); coughing, nasal discharge, and tachypnoea (73.19%); diarrhea, bloody diarrhea (53.86%); foot pad and nasal hyperkeratosis (43.00%); maculopapular rashes (41.55%); and nervous signs (19.81%).

### 3.3. Hematology and Blood Biochemistry

Blood hematological results indicated that CDV-infected dogs were anemia, leukopenia, neutrophilia, and thrombocytopenia. Furthermore, blood biochemistry results demonstrated that AST was slightly increased. However, our results found that BUN significantly rose in CDV-infected dogs and the urine protein/creatinine ratio was 18.30 (Table 2).

### 3.4. Nucleotide and Amino Acid Homology

**Figure 2:**
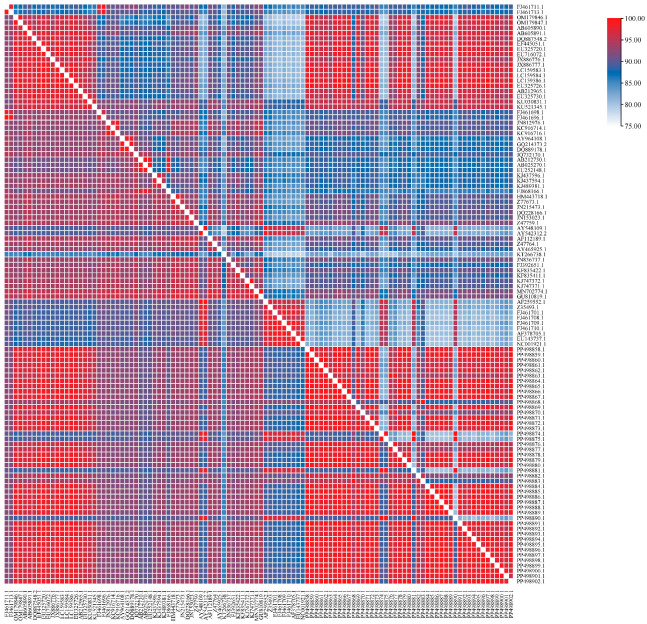
Nucleotide (below diagonal) and amino acid (above diagonal) homology of H gene of CDV isolated from dogs in MD of Vietnam.

In the current study, the partial H gene (613 bp) was utilized to amplify and analyze the nucleotide and amino acid homology. The results revealed that the nucleotide homology among the field CDV sequences in the MD region based on the partial H gene was 88.20–100%. The nucleotide homology between the field CDV sequences and other CDV sequences from dogs in Vietnam available on the GenBank database was high, accounting for 88.40–99.40%. The nucleotide homology between the field CDV sequences and Asia-1 genotype was 88.40–99.40%, and between the field CDV sequences and other reference CDV sequences in the world based on the database available on GenBank was 83.90–98.00%. The nucleotide homology between the field CDV sequences and the vaccine sequences based on the database available on GenBank was 87.50–98.30%. The field CDV sequences obtained from dogs in the MD region were a high amino acid homology based on the partial H gene, ranging from 85.90 to 100%. The amino acid homology between the field CDV sequences and other reference CDV sequences obtained from dogs in Vietnam was 85.90–100%, between the field CDV sequences and Asia-1 genotype was 79.10–100%, and between the field CDV sequences and other reference CDV sequences in the world based on the database available on GenBank was 79.60–95.60%. The amino acid homology between the field CDV sequences and the vaccine sequences based on the database available on GenBank was 79.60–96.70% (Figure 2).

**Figure 3:**
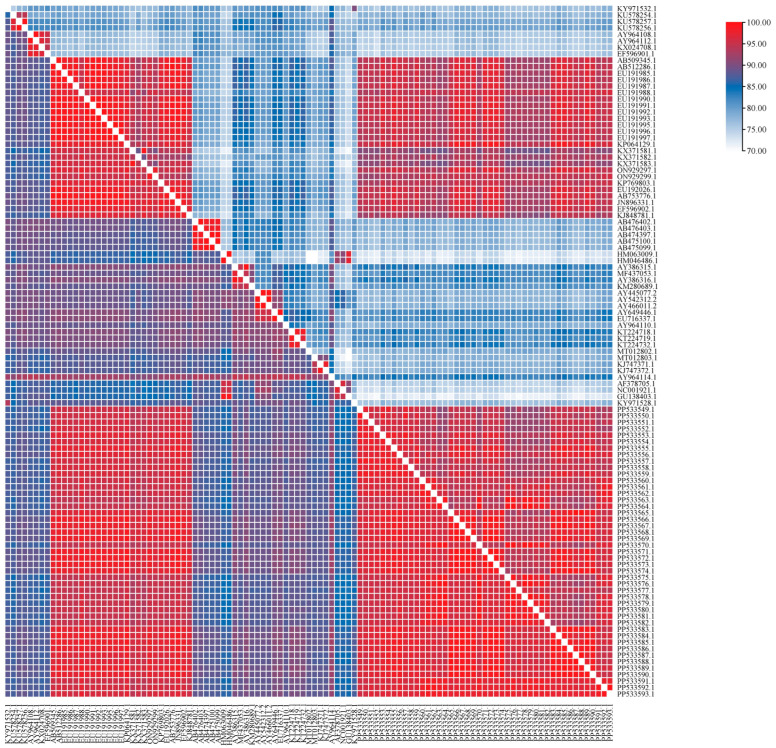
Nucleotide (below diagonal) and amino acid (above diagonal) homology of F gene of CDV isolated from dogs in MD of Vietnam.

A 594 bp fragment of the F gene was amplified and analyzed to determine nucleotide and amino acid homology. The results showed that the nucleotide homology based on the partial F gene of CDV among the field sequences obtained from dogs in the MD region was 94.90–100%. The nucleotide homology between the field CDV sequences and the Asia-1 genotype was 92.80–99.30%, and between the field CDV sequences and other reference CDV sequences in the world based on the database available on GenBank was 84.00–92.70%. The nucleotide homology between the field CDV sequences and the vaccine sequences was 84.00–86.40%. Moreover, the field CDV sequences were a fairly high amino acid homology, ranging from 90.80 to 100%. The amino acid homology between the field CDV sequences and the Asia-1 genotype was 88.30–98.90%, and between the field CDV sequences and other reference CDV sequences on GenBank was 71.00–85.70%. The amino acid homology between the field CDV sequences and the vaccine sequences was 70.50–77.10% (Figure 3).

### 3.5. Phylogenetic and Recombination Analysis

**Figure 4:**
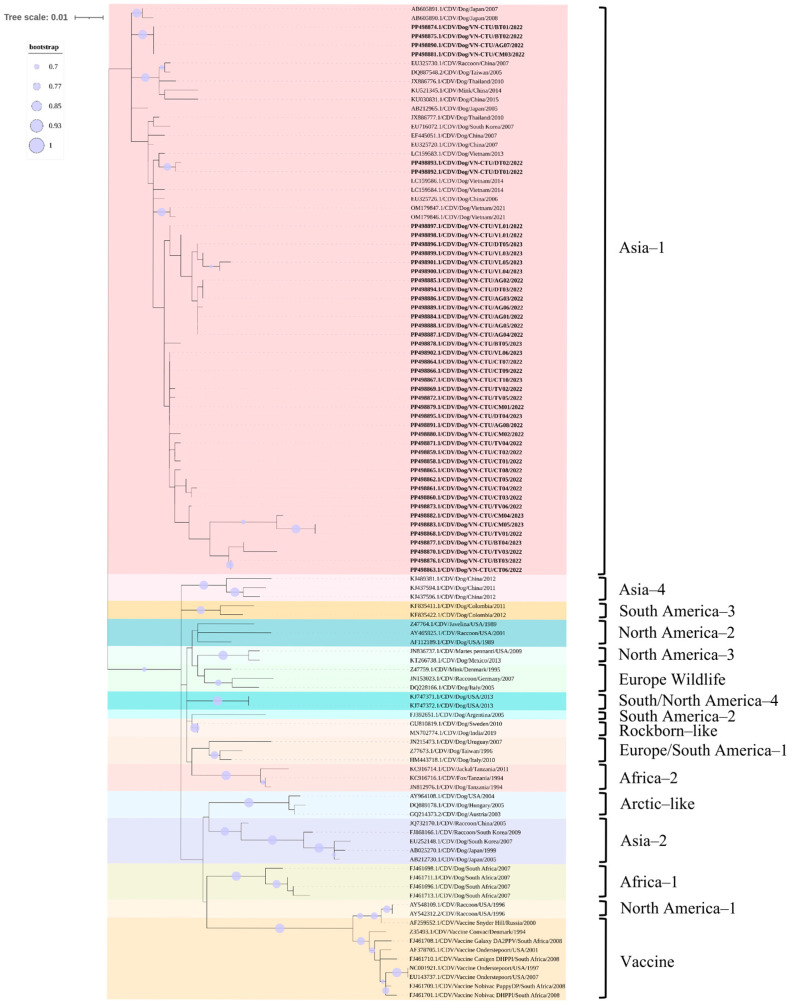
Phylogenetic tree of 45 field CDV sequences obtained from dogs raised in MD region and reference CDV sequences on GenBank based on nucleotide sequences of partial H gene. Field CDV sequences were highlighted. Bootstrap value over 70% was displayed in the branch of the tree.

The phylogenetic analysis according to the partial H gene of CDV indicated that the field CDV sequences obtained from dogs in the MD region were grouped in the same branch of Asia-1 genotype and were on the same branch as other CDV sequences previously obtained in the South of Vietnam, including three CDV sequences obtained from dogs in Ho Chi Minh City (GenBank accession number LC159583, LC159584, and LC159586) and the North of Vietnam, including two CDV sequences obtained from dogs in Ha Noi City (GenBank accession number OM179846 and OM179847). The field-derived CDV sequences were uniquely grouped into subgroups within an Asia-1 genotype cluster. In addition, the field CDV sequences obtained in the MD region were clustered in Asia-1 genotype based on previously published reference sequences on GenBank from neighboring countries in Asia, including China, Taiwan, Republic of Korea, Japan, and Thailand, and distanced from other genotypes circulating in dog populations worldwide. The Rockborn-like vaccine sequence was located in a separate group from the field CDV sequences and the America-1 genotype (Figure 4). However, the recombination results indicated that no genetic recombinant events were detected in this study based on the partial H gene analysis.

**Figure 5:**
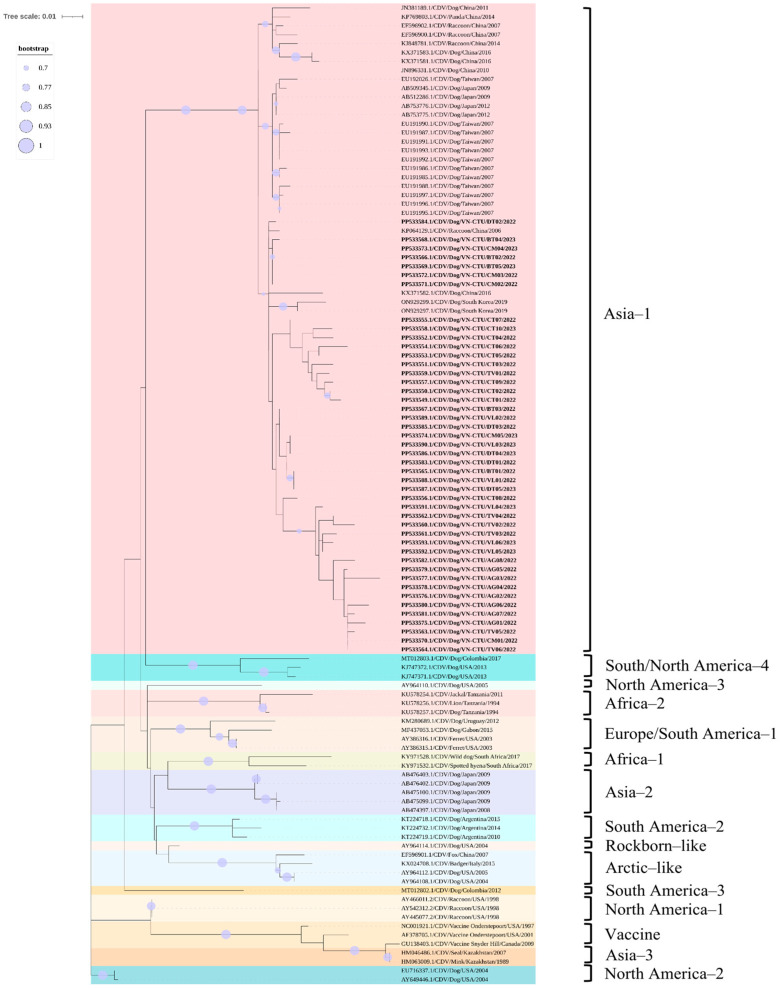
Phylogenetic tree of 45 field CDV sequences obtained from dogs raised in MD region and reference CDV sequences on GenBank based on nucleotide sequences of partial F gene. Field CDV sequences were highlighted. Bootstrap value over 70% was displayed in the branch of the tree.

Likewise, the phylogenetic analysis according to the partial F gene of CDV highlighted that the field CDV sequences obtained from dogs in the MD region were clustered in the same group of the Asia-1 genotype and were on the same branch as other CDV sequences based on the reference data available on GenBank that have been previously published in the neighboring countries in Asian, including China, Taiwan, Republic of Korea, and Japan. The field CDV sequences were divided into unique subgroups in each cluster within the same clade. However, the field CDV sequences were ungrouped with other genotypes circulating in dog populations globally, such as America-2, Arctic-like, Europe-1/South America-1, North America-3, South America-2, and South America/North America-4. The Rockborn-like vaccine sequence was located in a separate group compared to the field CDV sequences (Figure 5). Furthermore, the recombination events were not found in the current study based on the partial F gene analysis.

### 3.6. Potential N-Linked Glycosylation Sites

According to the prediction of the potential N-linked glycosylation sites of the H protein of the field-derived CDV sequences in the MD region, the results showed that two potential N-linked glycosylation sites were predicted, at positions 391 and 422 (Table S3). In addition, our results indicated that there were four potential N-linked glycosylation sites of the F protein, at positions 62, 108, 173, and 179 (Table S4).

## 4. Discussion

CDV has been recognized as a serious cause of death in domestic dog populations and wild carnivores worldwide, following rabies [31,32]. CDV infection in dogs is a systemic disease characterized by respiratory, gastrointestinal, and neurological signs. Additionally, naturally infected dogs often exhibit alterations in physiological and biochemical blood parameters [33,34]. The clinical signs can vary significantly and have been influenced by factors such as the age and immune status of dogs [2,32]. Although both live attenuated and recombinant vaccines against CDV are widely available and have been continually improved, outbreaks of CD continue to be reported in various geographical regions of the world [13,16,35,36,37,38]. It highlights the ongoing need for region-specific studies to better understand the epidemiological, clinical, and molecular characteristics of CDV infections to inform more effective strategies for disease control and prevention on a global scale [13,36,37].

The MD region includes one centrally governed city (Can Tho City) and some provinces, including An Giang, Ben Tre, Ca Mau, Dong Thap, Tra Vinh, and Vinh Long. Among these, Can Tho City is an economic, cultural, and social center of the entire region with a high rate of urbanization [39], and it may impact the understanding of the owners on the diseases in dogs and increase access to better care for dogs in Can Tho City compared to other provinces in the region. According to the results, puppies (<6 months old) were at higher risk of CD than other dog groups (*p* < 0.01). Similarly, previous studies indicated that age was a risk factor for CD (*p* < 0.05), and that puppies were more susceptible to CDV than adult dogs [40,41]. Age may affect the development and decline of the immune system. Young animals of most animal species depend on passively acquired immunity from their mother through colostrum. Therefore, young animals were at higher risk of infection than adults [42,43,44]. In addition, free-roaming dogs were at higher risk of infection than confined dogs (*p* < 0.01). The results of the current study were similar to the earlier study, where spatial analysis showed that CDV exposure was more common in free-roaming dogs (*p* < 0.05) [41]. Similarly, the results of the prior study indicated that the free movement of rural dogs increased the risk of exposure to CDV through exposure to pathogens. In addition, the free movement of the rural dogs among villages was also a cause of CD transmission in Chile [45]. Therefore, the free-roaming situation was considered a risk factor for CD transmission. Furthermore, unvaccinated dogs were at a higher risk of CD infection than dogs in other groups (*p* < 0.01). According to the earlier investigation, vaccination status was a risk factor associated with CD in Poland (*p* < 0.01) [46]. Annual vaccination is an important strategy to protect dogs from CDV [38]. According to the manufacturer’s recommendations, fully and regularly vaccinated dogs have a better immune response to CDV than dogs that have not been vaccinated or have not been fully vaccinated [47]. Moreover, several prior studies have shown that puppies may be susceptible to CDV due to the failure of the booster vaccination program, emphasizing the importance of a timely and complete vaccination program in puppies [48,49]. In contrast, the current results additionally demonstrated that there was no relationship between breed and sex of dogs and the CD (*p* > 0.05). Indeed, the previous studies found no association between the breed and sex of CDV-infected dogs and CDV positivity rate (*p* > 0.05) [50,51,52].

In dogs, CDV can affect the host in several internal systems such as the digestive, respiratory, and central nervous systems [2,53]. The weak immune response of CDV-infected dogs in the early stages leads to the appearance of nonspecific clinical signs of the disease, such as fever, depression, anorexia, and vomiting. Clinically, dogs in the study exhibited the clinical signs of CDV infection, including ocular discharge, conjunctivitis, and respiratory sings (coughing, sneezing, green nasal discharge). Moreover, the typical clinical signs of the digestive tract often manifest clearly in CDV-infected dogs, including diarrhea and/or bloody diarrhea. In addition, this study suggested that foot pad and nasal hyperkeratosis, and maculopapular rashes can be considered a typical clinical sign of CDV infection in dogs. On the other hand, the rate of nervous signs was quite low, accounting for only 19.81% of all dogs infected with CDV. The reason may be that nervous signs have appeared in the late stages and in prolonged cases [54]. Central nervous system infection is the most serious complication of CD, leading to a variety of neurological disorders, and dogs with neurological signs often have a poor prognosis [55,56,57]. Overall, the suspected dogs in the current study exhibited the common clinical signs of CDV infection.

Herein, the results of hematology and blood biochemistry demonstrated that there were abnormal changes in the CDV-infected dogs. The RBC, HGB, MCV, MCH, and MCHC of CDV-infected dogs were reduced, and these findings indicated that CDV may cause anemia in infected dogs. The decrease in RBC of CDV-infected dogs may be related to intestinal hemorrhage [58]. Furthermore, the persistence of CDV in the bone marrow may cause erythroid hypoplasia, and thus may be the cause of anemia in infected dogs. Indeed, CDV infection has been connected to bone marrow pathology, leading to anemia in the host [33,59,60]. Other possible causes of the anemia observed in CDV-infected dogs may be the production of inflammatory mediators, which may inhibit erythropoiesis and also shorten the lifespan of erythrocytes [33,59]. In addition, leukopenia and neutrophilia in CDV-infected dogs were reported in the current study. CDV replication in the lymph nodes may be responsible for the primary leukopenia caused by CDV-induced lymphocytic damage and consequent leukopenia in the blood due to excessive leukocyte recruitment in a short time and a lack of leukocyte production in the lymph nodes. Furthermore, subsequent to the initial leukopenia, dogs infected with CDV have developed lymphocytosis and thus leukocytosis. Therefore, both leukopenia and leukocytosis have been considered CDV features. In early CDV infections, lymphopenia can be predicted; meanwhile, the late CDV infections have been characterized by lymphocytosis [33,59,60]. On the other hand, thrombocytopenia was observed in the CDV-infected dogs in the current study. Persistent CDV infection in the bone marrow may cause thrombocytopenia in infected dogs, as CDV is a chronic infection known to induce bone marrow pathology, leading to thrombocytopenia [33,59].

Furthermore, the results of the present study showed that AST was slightly increased, whereas there was no change in ALT in the CDV-positive dogs. These results suggest that blood biochemical parameters have a weak relationship with the viral infection; however, it mainly depends on the age, physical condition, and internal diseases of the dog. The increase or decrease of ALT and AST in the blood of dogs was a non-specific sign of CD. Prior studies have shown that there was no significant change in ALT and AST levels in the blood of CDV-infected dogs compared to dogs in the control group [33,60]. The change in ALT and AST levels in the blood of CDV-infected dogs may be affected by age and other internal diseases. Therefore, the change in these blood biochemical parameters was not a specific sign of CDV infection [33]. Additionally, the results of the current study showed that BUN in the blood of the infected dogs significantly increased. Similarly, the results of the past study exhibited that dogs infected with CDV were a higher BUN level than the control group [59]. The increase in BUN levels may be due to dehydration subsequent to prolonged diarrhea in dogs infected with CD [59]. Moreover, the *Morbillivirus* genus has been reported to cause kidney damage and affect kidney filtration function [34]. BUN is known as a major nitrogenous waste product from protein metabolism. The variation in BUN levels in the blood may be affected by impaired function of the kidneys and liver of the host, which can increase BUN levels. However, these changes were also considered non-specific manifestations of CDV infection and vary depending on each infected dog [33]. In addition, the results of the current study showed that there was no change in creatinine levels in the blood of CDV-infected dogs. Similarly, a previous study found that there was no change in the creatinine level in dogs infected with CDV compared to the control group [60]. However, the increase or decrease of the creatinine level in infected dogs depended on the levels of kidney damage caused by CDV to the host [34].

CDV is a non-segmented, negative-sense single-stranded RNA virus containing a genome encoding six structural and two non-structural proteins. Among these proteins, the H protein is a component of the envelope glycoprotein spikes on the virion and plays an important role in host cell entry by binding to cellular receptors such as the signaling lymphocyte activation molecule (SLAM, CD150) or Nectin-4 [61,62]. In immunological roles, the H glycoprotein is the main target for neutralizing antibodies and is therefore under strong selective pressure from the host immune response, exhibiting the highest sequence variability among CDV genes [63,64].

Due to the high variability, the H gene is widely used as a target for phylogenetic studies and classification of CDV into distinct genotypes based on the identity between gene/protein sequences [16,31,36,62,64]. Besides the H gene, the F gene of CDV has a prominent role in the invasion of the virus into host cells by fusing the viral virion envelope and the outer membrane of the host cell [8,65]. Analogous to the H gene, the genetic variations in the F gene may emerge as a consequence of mutation and natural selection pressures. In fact, the genetic variations in the H and F genes of CDV may be due to the reassortment of field CDV strains and vaccine strains, or interactions between CDV and the host [31,66]. The present study, therefore, utilized the partial H and F genes of CDV to demonstrate the molecular characteristics of the circulating CDV sequences in the MD region of Vietnam.

In this document, the results revealed a high nucleotide and amino acid homology among the field CDV sequences in the MD region (up to 100%) as well as between the field CDV sequences and the Asia-1 genotype (over 99.00%) based on the H and F genes. However, there was a lower homology among the field CDV sequences, other genotypes worldwide, and the vaccine sequence based on the available database of the H and F genes on GenBank compared to the Asia-1 genotype. Correlation with the nucleotide and amino acid homology of the partial H and F genes, the phylogenetic analysis results exhibited that all field CDV sequences were clustered into the same clade with the Asia-1 genotype, and formed a separate group from other CDV genotypes circulating worldwide, as well as from the vaccine sequence based on the available database on GenBank. Furthermore, the present study displayed that the genetic recombinant events were not found in the partial H and F genes.

This study collected samples of several vaccinated dogs, and the partial H and F sequences were generated. However, phylogenetic data excluded the involvement of vaccine-derived strains exhibiting residual virulence. Accordingly, CD occurred in vaccinated dogs that likely stemmed from improper vaccine handling, poor immune response of the host to the vaccine, or antigenic divergence in field strains compromising vaccine efficacy [12,22,37]. In fact, even though CD was first documented in Vietnam around the 1950s, the first molecular report of CDV was published in 2009 and indicated that the field-derived CDV sequences collected from dogs in the North of Vietnam belonged to the America-1 genotype [19]. Following this, the study on the molecular characteristics of the field CDV sequences collected from dogs in Ho Chi Minh City based on the H gene showed that the field CDV sequences were grouped into the Asia-1 genotype and were in the same branch as CDV sequences circulating in other Asian countries, including Taiwan, China, Republic of Korea, Japan, and Thailand, and different from previous studies in Vietnam [20]. In 2022, novel clades of the Asia-1 genotype were first demonstrated from the infected dogs in the North of Vietnam [21]. Therefore, at least three genotype variables of CDV were circulating in Vietnam, including America-1, Asia-1, and/or novel clades of Asia-1 genotype based on the H and/or P genes, and these genotypes showed similarities in genetic origin between countries in the same neighboring geographical area [19,20,21]. The present study once again confirmed that Asia-1 genotype should be noted as the main genotype circulating in dogs in Vietnam.

However, this study encountered a limitation; only forty-five partial H and F genes were used to identify the molecular characteristics of the circulating CDV sequences in the MD region. The use of non-random sequence selection based on available data may introduce selection bias. However, the sequences were chosen to reflect a variety of epidemiological variables; thus, the results were intended to balance the completeness of epidemiological and molecular data. Further studies should be promoted to address this limitation of the current study.

To date, the H protein is widely used to assess genetic variation in the field strains of CDV, with site-specific signatures of variation providing insights into the evolutionary trajectory of the virus [62,67]. Approximately eight to ten potential N-linked glycosylation sites (19–21, 149–151, 309–311, 339–341, 391–393, 422–424, 456–458, 542–544, 584–586, 587–589, 603–605) are highly conserved in the H protein of CDV [4,5,6]. Furthermore, the number of potential N-linked glycosylation sites in the H protein is associated with the virulence of CDV [68]. In recent years, several studies have attempted to address the role of the N-glycosylation pattern of the H protein in the virulence of CDV [21,37,62]. These glycosylation patterns may influence neutralizing epitopes, potentially disrupting antigenic sites critical for host immune protection [37,69]. In the F gene, four potential N-linked glycosylation sites were identified. Three of these sites were located in the F2 region at amino acid positions 141–143, 173–175, and 179–181; meanwhile, the remaining site was located in the F1 region at positions 517–519 [7,8,9,13]. Current hypotheses suggest that reduced potential N-linked glycosylation may mitigate CDV pathogenicity; meanwhile, increased glycosylation may enhance immune evasion mechanisms, potentially undermining vaccine efficacy [13,68]. According to the present findings, two potential N-linked glycosylation sites were predicted in the partial H gene and four in the partial F protein. However, the analysis of the partial H and F genes from the field-derived CDV sequences revealed no extra potential N-linked glycosylation sites. This result suggests that reliance on partial sequences may overlook other potential N-linked glycosylation sites that could be present in different positions in the genome of CDV. Therefore, additional investigations on the full-length genome of CDV is warranted to clearly indicate the potential N-linked glycosylation sites and explore the role of these glycosylation in CDV pathogenesis.

## 5. Conclusions

The findings of the current study first exhibited the epidemiological and clinical features of CDV infection in clinically suspected dogs raised in seven provinces/cities in the MD region of Vietnam. In addition, our findings once again confirmed that the Asia-1 genotype represents the predominant CDV strain circulating in dog populations in Vietnam. These findings could contribute to developing the control strategies against CDV in Vietnam in the future. Further discussions on broader data in wildlife hosts and various regions should be encouraged to clearly elucidate viral diversity, transmission patterns, and geographic spread of CDV in Vietnam.

## Figures and Tables

**Table 1:** Summary of demographic characteristics of CDV-positive dogs and risk factors.

Category	No. of Positive Dogs/Total	Percentage (%)	OR (95% CI)	*p*-Value
Age				
<6 months	202/1729	11.68 ^a^	Ref.	0.001
6 months–2 years	38/1662	2.29 ^c^	5.65 (3.97–8.05)	
2–5 years	49/1694	2.89 ^c^	4.44 (3.22–6.11)	
>5 years	125/1602	7.80 ^b^	1.56 (1.24–1.98)	
Breed				
Pure/cross	189/2836	6.66	0.88 (0.72–1.07)	0.20
Local	225/3851	5.84		
Sex				
Male	225/3431	6.56	1.14 (0.93–1.39)	0.20
Female	189/3256	5.80		
Roaming status				
Free-roaming	272/3735	7.28 ^a^	1.51 (1.23–1.87)	0.001
Confined	142/2952	4.81 ^b^		
Vaccination status				
No	298/2180	13.67 ^a^	Ref.	0.001
One dose	95/2087	4.55 ^b^	3.32 (2.61–4.22)	
Two doses	21/2420	0.87 ^c^	18.09 (11.57–28.27)	

^a–c^ Within a group, mean without a common superscript differs (*p* < 0.01).

**Table 2:** Hematology and blood biochemistry results.

Category	Reference	CDV-Infected Dogs (Mean ± SE)	Min–Max
RBC (10^6^/mm^3^)	5–10	4.02 ± 0.06	1.10–5.99
HCT (%)	30–45	31.38 ± 0.28	20.00–39.80
MCV (fL)	60–77	52.48 ± 0.80	21.10–74.90
HGB (g/dL)	8–15	8.38 ± 0.06	6.10–10.90
MCH (Pg)	19.50–24.50	14.94 ± 0.19	10.00–22.50
MCHC (%)	32–36	28.20 ± 0.09	22.60–35.00
WBC (10^3^/mm^3^)	5.50–19.50	4.45 ± 0.08	1.05–7.60
Neutrophil (%)	35–75	78.33 ± 0.18	75.00–97.39
Lymphocyte (%)	20–55	27.21 ± 0.08	25.00–40.80
Monocyte (%)	1–4	2.72 ± 0.10	1.01–13.06
Eosinophil (%)	2–12	6.54 ± 0.04	3.46–7.98
Basophil (%)	0–1	0.43 ± 0.003	0.30–0.66
PLT (10^3^/mm^3^)	300–700	283.58 ± 5.06	101.00–499.40
AST (U/L)	8.90–48.50	54.69 ± 1.15	28.00–159.00
ALT (U/L)	8.20–57.30	56.44 ± 0.94	22.00–114.00
BUN (mg/dL)	3.10–9.20	27.46 ± 0.25	20.10–39.90
Creatinine (mg/dL)	0.50–1.60	1.50 ± 0.01	1.00–1.90

## Data Availability

The data presented in this study are available within the article. Raw data supporting this study are available from the corresponding author.

## Supplementary Materials

The following supporting information can be downloaded at: https://www.mdpi.com/article/10.3390/v17060781/s1, Table S1: Detailed information about CDV-sequencing samples in the current study; Table S2: Detailed information about reference CDV sequences based on GenBank database; Table S3: Potential N-linked glycosylation sites of H gene of CDV sequences collected from dogs raised in MD region of Vietnam; Table S4: Potential N-linked glycosylation sites of F gene of CDV sequences collected from dogs raised in MD region of Vietnam.

## References

[1] Sidhu M.S., Husar W., Cook S.D., Dowling P.C., Udem S.A. (1993). Canine distemper terminal and intergenic non-protein coding nucleotide sequences: Completion of the entire CDV genome sequence. Virology.

[2] Rendon-Marin S., da Fontoura Budaszewski R., Canal C.W., Ruiz-Saenz J. (2019). Tropism and molecular pathogenesis of canine distemper virus. Virol. J..

[3] Lamb R.A. (1993). Paramyxovirus fusion: A hypothesis for changes. Virology.

[4] Zhao J.-J., Yan X.-J., Chai X.-L., Martella V., Luo G.-L., Zhang H.-L., Gao H., Liu Y.-X., Bai X., Zhang L. (2010). Phylogenetic analysis of the haemagglutinin gene of canine distemper virus strains detected from breeding foxes, raccoon dogs and minks in China. Vet. Microbiol..

[5] Tan B., Wen Y.-J., Wang F.-X., Zhang S.-Q., Wang X.-D., Hu J.-X., Shi X.-C., Yang B.-C., Chen L.-Z., Cheng S.-P. (2011). Pathogenesis and phylogenetic analyses of canine distemper virus strain ZJ7 isolate from domestic dogs in China. Virol. J..

[6] Liu Y., Liu C., Liu W., Wu H., Ding H., Cao Y., Spibey N., Wang L., He W., Hao L. (2019). Isolation and sequence analysis of the complete H gene of canine distemper virus from domestic dogs in Henan Province, China. Arch. Virol..

[7] Li W., Li T., Liu Y., Gao Y., Yang S., Feng N., Sun H., Wang S., Wang L., Bu Z. (2014). Genetic characterization of an isolate of canine distemper virus from a Tibetan Mastiff in China. Virus Genes.

[8] Romanutti C., Calderón M.G., Keller L., Mattion N., La Torre J. (2016). RT-PCR and sequence analysis of the full-length fusion protein of canine distemper virus from domestic dogs. J. Virol. Methods.

[9] Freitas L.A., Leme R.A., Saporiti V., Alfieri A.A., Alfieri A.F. (2019). Molecular analysis of the full-length F gene of Brazilian strains of canine distemper virus shows lineage co-circulation and variability between field and vaccine strains. Virus Res..

[10] Wipf A., Perez-Cutillas P., Ortega N., Huertas-López A., Martínez-Carrasco C., Candela M. (2025). Geographical Distribution of Carnivore Hosts and Genotypes of Canine Distemper Virus (CDV) Worldwide: A Scoping Review and Spatial Meta-Analysis. Transbound. Emerg. Dis..

[11] Martella V., Blixenkrone-Møller M., Elia G., Lucente M.S., Cirone F., Decaro N., Nielsen L., Banyai K., Carmichael L., Buonavoglia C. (2011). Lights and shades on an historical vaccine canine distemper virus, the Rockborn strain. Vaccine.

[12] Radtanakatikanon A., Keawcharoen J., taya Charoenvisal N., Poovorawan Y., Prompetchara E., Yamaguchi R., Techangamsuwan S. (2013). Genotypic lineages and restriction fragment length polymorphism of canine distemper virus isolates in Thailand. Vet. Microbiol..

[13] da Costa V.G., Saivish M.V., de Oliveira P.G., Silva-Júnior A., Moreli M.L., Krüger R.H. (2021). First complete genome sequence and molecular characterization of Canine morbillivirus isolated in Central Brazil. Sci. Rep..

[14] Saltık H.S., Atlı K. (2023). Approaches to identify canine distemper virus with neurological symptoms on the basis of molecular characterization of hemagglutinin and fusion genes. Virus Genes.

[15] Lee M.-S., Tsai K.-J., Chen L.-H., Chen C.-Y., Liu Y.-P., Chang C.-C., Lee S.-H., Hsu W.-L. (2010). The identification of frequent variations in the fusion protein of canine distemper virus. Vet. J..

[16] Piewbang C., Radtanakatikanon A., Puenpa J., Poovorawan Y., Techangamsuwan S. (2019). Genetic and evolutionary analysis of a new Asia-4 lineage and naturally recombinant canine distemper virus strains from Thailand. Sci. Rep..

[17] Panzera Y., Sarute N., Iraola G., Hernández M., Pérez R. (2015). Molecular phylogeography of canine distemper virus: Geographic origin and global spreading. Mol. Phylogenet. Evol..

[18] Rivera-Martínez A., Rodríguez-Alarcón C.A., Adame-Gallegos J.R., Laredo-Tiscareño S.V., de Luna-Santillana E.d.J., Hernández-Triana L.M., Garza-Hernández J.A. (2024). Canine Distemper Virus: Origins, Mutations, Diagnosis, and Epidemiology in Mexico. Life.

[19] Lan N.T., Yamaguchi R., Kien T.T., Hirai T., Hidaka Y., Nam N.H. (2009). First isolation and characterization of canine distemper virus in Vietnam with the immunohistochemical examination of the dog. J. Vet. Med. Sci..

[20] Van Nguyen D., Suzuki J., Minami S., Yonemitsu K., Nagata N., Kuwata R., Shimoda H., Vu C.K., Truong T.Q., Maeda K. (2017). Isolation and phylogenetic analysis of canine distemper virus among domestic dogs in Vietnam. J. Vet. Med. Sci..

[21] Truong Q.L., Duc H.M., Anh T.N., Thi Y.N., Van T.N., Thi P.H., Thu H.N.T., Thi L.N. (2022). Isolation and genetic characterization of canine distemper virus in domestic dogs from central and northern provinces in Vietnam. Res. Vet. Sci..

[22] Van T.M., Le T.Q., Tran B.N. (2023). Phylogenetic characterization of the canine distemper virus isolated from veterinary clinics in the Mekong Delta, Vietnam. Vet. World.

[23] Gámiz C., Martella V., Ulloa R., Fajardo R., Quijano-Hernandéz I., Martínez S. (2011). Identification of a new genotype of canine distemper virus circulating in America. Vet. Res. Commun..

[24] Hall T.A. (1999). BioEdit: A user-friendly biological sequence alignment editor and analysis program for Windows 95/98/NT. Nucl. Acids Symp. Ser..

[25] Kumar S., Stecher G., Tamura K. (2016). MEGA7: Molecular evolutionary genetics analysis version 7.0 for bigger datasets. Mol. Biol. Evol..

[26] Letunic I., Bork P. (2024). Interactive Tree of Life (iTOL) v6: Recent updates to the phylogenetic tree display and annotation tool. Nucleic Acids Res..

[27] Martin D.P., Murrell B., Golden M., Khoosal A., Muhire B. (2015). RDP4: Detection and analysis of recombination patterns in virus genomes. Virus Evol..

[28] Lole K.S., Bollinger R.C., Paranjape R.S., Gadkari D., Kulkarni S.S., Novak N.G., Ingersoll R., Sheppard H.W., Ray S.C. (1999). Full-length human immunodeficiency virus type 1 genomes from subtype C-infected seroconverters in India, with evidence of intersubtype recombination. J. Virol..

[29] Gupta R., Brunak S. (2002). Prediction of glycosylation across the human proteome and the correlation to protein function. Pac. Symp. Biocomput..

[30] Chen C., Wu Y., Li J., Wang X., Zeng Z., Xu J., Liu Y., Feng J., Chen H., He Y. (2023). TBtools-II: A “one for all, all for one” bioinformatics platform for biological big-data mining. Mol. Plant.

[31] Loots A.K., Mokgokong P.S., Mitchell E., Venter E.H., Kotze A., Dalton D.L. (2018). Phylogenetic analysis of canine distemper virus in South African wildlife. PLoS ONE.

[32] Deem S.L., Spelman L.H., Yates R.A., Montali R.J. (2000). Canine distemper in terrestrial carnivores: A review. J. Zoo Wildl. Med..

[33] Buragohain M., Goswani S., Kalita D. (2017). Clinicopathological findings of canine distemper virus infection in dogs. J. Entomol. Zool. Stud..

[34] Silva M.d.L.e., Silva G.E.B., Borin-Crivellenti S., Alvarenga A.W.O., Aldrovani M., Braz L.A.d.N., Aoki C., Santana A.E., Pennacchi C.S., Crivellenti L.Z. (2022). Renal abnormalities caused by canine distemper virus infection in terminal patients. Front. Vet. Sci..

[35] Riley M.C., Wilkes R.P. (2015). Sequencing of emerging canine distemper virus strain reveals new distinct genetic lineage in the United States associated with disease in wildlife and domestic canine populations. Virol. J..

[36] Wang H., Guo H., Hein V.G., Xu Y., Yu S., Wang X. (2023). The evolutionary dynamics history of canine distemper virus through analysis of the hemagglutinin gene during 1930–2020. Eur. J. Wildl. Res..

[37] Bhatt M., Rajak K., Chakravarti S., Yadav A., Kumar A., Gupta V., Chander V., Mathesh K., Chandramohan S., Sharma A. (2019). Phylogenetic analysis of haemagglutinin gene deciphering a new genetically distinct lineage of canine distemper virus circulating among domestic dogs in India. Transbound. Emerg. Dis..

[38] Wilkes R.P. (2022). Canine distemper virus in endangered species: Species jump, clinical variations, and vaccination. Pathogens.

[39] Ngo-Duc T. (2014). Climate change in the coastal regions of Vietnam. Coastal Disasters and Climate Change in Vietnam.

[40] Lechner E.S., Crawford P.C., Levy J.K., Edinboro C.H., Dubovi E.J., Caligiuri R. (2010). Prevalence of protective antibody titers for canine distemper virus and canine parvovirus in dogs entering a Florida animal shelter. J. Am. Vet. Med. Assoc..

[41] McDermott I., Gilbert M., Shah M.K., Sadaula A., Anderson N.E. (2023). Seroprevalence of canine distemper virus (CDV) in the free-roaming dog (*Canis familiaris*) population surrounding Chitwan National Park, Nepal. PLoS ONE.

[42] Greeley E.H., Spitznagel E., Lawler D.F., Kealy R.D., Segre M. (2006). Modulation of canine immunosenescence by life-long caloric restriction. Vet. Immunol. Immunopathol..

[43] Schultz R., Thiel B., Mukhtar E., Sharp P., Larson L. (2010). Age and long-term protective immunity in dogs and cats. J. Comp. Pathol..

[44] Day M. (2007). Immune system development in the dog and cat. J. Comp. Pathol..

[45] Acosta-Jamett G., Chalmers W., Cunningham A., Cleaveland S., Handel I., Bronsvoort B.d. (2011). Urban domestic dog populations as a source of canine distemper virus for wild carnivores in the Coquimbo region of Chile. Vet. Microbiol..

[46] Jóźwik A., Frymus T., Mizak B., Rzeżutka A. (2004). Antibody titres against canine distemper virus in vaccinated and unvaccinated dogs. J. Vet. Med. Ser. B.

[47] Eghafona N., Jacob J., Yah S. (2007). Evaluation of post-vaccination immunity to canine distemper and parvoviruses in Benin City, Nigeria. Afr. J. Biotechnol..

[48] Kapil S., Yeary T.J. (2011). Canine distemper spillover in domestic dogs from urban wildlife. Vet. Clin. N. Am. Small Anim. Pract..

[49] Wilson S., Siedek E., Thomas A., King V., Stirling C., Plevová E., Salt J., Sture G. (2014). Influence of maternally-derived antibodies in 6-week old dogs for the efficacy of a new vaccine to protect dogs against virulent challenge with canine distemper virus, adenovirus or parvovirus. Trials Vaccinol..

[50] Dorji T., Tenzin T., Tenzin K., Tshering D., Rinzin K., Phimpraphai W., de Garine-Wichatitsky M. (2020). Seroprevalence and risk factors of canine distemper virus in the pet and stray dogs in Haa, western Bhutan. BMC Vet. Res..

[51] Costa V.G.d., Saivish M.V., Rodrigues R.L., Lima Silva R.F.d., Moreli M.L., Krüger R.H. (2019). Molecular and serological surveys of canine distemper virus: A meta-analysis of cross-sectional studies. PLoS ONE.

[52] Dong B., Zhang X., Wang J., Zhang G., Li C., Wei L., Lin W. (2021). A meta-analysis of cross-sectional studies on the frequency and risk factors associated with canine morbillivirus infection in China. Microb. Pathog..

[53] Greene C.E. (2006). Infectious Diseases of the Dog and Cat.

[54] Amude A., Alfieri A., Alfieri A. (2007). Clinicopathological findings in dogs with distemper encephalomyelitis presented without characteristic signs of the disease. Res. Vet. Sci..

[55] Beineke A., Baumgärtner W., Wohlsein P. (2015). Cross-species transmission of canine distemper virus—An update. One Health.

[56] Gebara C., Wosiacki S., Negrao F., De Oliveira D., Beloni S., Alfieri A., Alfieri A. (2004). Detection of canine distemper virus nucleoprotein gene by RT-PCR in urine of dogs with distemper clinical signs. Arq. Bras. Med. Vet. E Zootec..

[57] Carvalho O.V., Botelho C.V., Ferreira C.G.T., Scherer P.O., Soares-Martins J.A.P., Almeida M.R., Silva Júnior A. (2012). Immunopathogenic and neurological mechanisms of canine distemper virus. Adv. Virol..

[58] Salem N. (2014). Canine viral diarrhea: Clinical, hematologic and biochemical alterations with particular reference to in-clinic rapid diagnosis. Glob. Vet..

[59] Saaed M.M., Al-Obaidi Q.T. (2021). Clinical, Hematological and Some Biochemical Changes In Dogs Infected With Canine Distemper. J. Agric. Vet. Sci..

[60] Willi B., Spiri A.M., Meli M.L., Grimm F., Beatrice L., Riond B., Bley T., Jordi R., Dennler M., Hofmann-Lehmann R. (2015). Clinical and molecular investigation of a canine distemper outbreak and vector-borne infections in a group of rescue dogs imported from Hungary to Switzerland. BMC Vet. Res..

[61] Noyce R.S., Delpeut S., Richardson C.D. (2013). Dog nectin-4 is an epithelial cell receptor for canine distemper virus that facilitates virus entry and syncytia formation. Virology.

[62] Ke G.-M., Ho C.-H., Chiang M.-J., Sanno-Duanda B., Chung C.-S., Lin M.-Y., Shi Y.-Y., Yang M.-H., Tyan Y.-C., Liao P.-C. (2015). Phylodynamic analysis of the canine distemper virus hemagglutinin gene. BMC Vet. Res..

[63] Harder T., Osterhaus A. (1997). Canine distemper virus—a morbillivirus in search of new hosts?. Trends Microbiol..

[64] Mochizuki M., Hashimoto M., Hagiwara S., Yoshida Y., Ishiguro S. (1999). Genotypes of canine distemper virus determined by analysis of the hemagglutinin genes of recent isolates from dogs in Japan. J. Clin. Microbiol..

[65] Evans S.A., Belsham G.J., Barrett T. (1990). The role of the 5′ nontranslated regions of the fusion protein mRNAs of canine distemper virus and rinderpest virus. Virology.

[66] Duque-Valencia J., Diaz F.J., Ruiz-Saenz J. (2019). Phylogenomic analysis of two co-circulating canine distemper virus lineages in Colombia. Pathogens.

[67] Fuques E., Tomás G., Grecco S., Condon E., Techera C., Marandino A., Sarute N., Aldaz J., Enciso J., Benech A. (2022). Origin and spreading of canine morbillivirus in South America. Virus Res..

[68] Sawatsky B., von Messling V. (2010). Canine distemper viruses expressing a hemagglutinin without N-glycans lose virulence but retain immunosuppression. J. Virol..

[69] Iwatsuki K., Miyashita N., Yoshida E., Gemma T., Shin Y.-S., Mori T., Hirayama N., Kai C., Mikami T. (1997). Molecular and phylogenetic analyses of the haemagglutinin (H) proteins of field isolates of canine distemper virus from naturally infected dogs. J. Gen. Virol..

