# Supplementary material for: Epidemiological, Clinical, and Molecular Insights into Canine Distemper Virus in the Mekong Delta Region of Vietnam
Source: Viruses. 2025 May 29;17(6):781. doi: 10.3390/v17060781 (PMC12197358; doi:10.3390/v17060781)
Supplement: Supplementary file 1 [file viruses-17-00781-s001.zip › Table S1.pdf]

Table S1: Detailed information about CDV-sequencing samples in the current study.

No.	GenBank accession number		Sample	Province/City	Collected year	Age (month)	Sex	Vaccination status	Clinical signs
	H gene	F gene							
1	PP498858	PP533549	CDV/VN-CTU/CT01	Can Tho City	2022	6	Male	No	RS, NS
2	PP498859	PP533550	CDV/VN-CTU/CT02	Can Tho City	2022	4	Female	Yes	GS
3	PP498860	PP533551	CDV/VN-CTU/CT03	Can Tho City	2022	12	Female	Yes	RS
4	PP498861	PP533552	CDV/VN-CTU/CT04	Can Tho City	2022	8	Female	No	RS, GS, NS
5	PP498862	PP533553	CDV/VN-CTU/CT05	Can Tho City	2022	24	Male	No	RS
6	PP498863	PP533554	CDV/VN-CTU/CT06	Can Tho City	2022	84	Male	No	RS, GS
7	PP498864	PP533555	CDV/VN-CTU/CT07	Can Tho City	2022	11	Female	No	RS, GS
8	PP498865	PP533556	CDV/VN-CTU/CT08	Can Tho City	2022	48	Female	No	RS
9	PP498866	PP533557	CDV/VN-CTU/CT09	Can Tho City	2022	36	Male	Yes	GS
10	PP498867	PP533558	CDV/VN-CTU/CT10	Can Tho City	2023	12	Male	Yes	GS
11	PP498868	PP533559	CDV/VN-CTU/TV01	Tra Vinh Province	2022	3	Male	No	RS, GS
12	PP498869	PP533560	CDV/VN-CTU/TV02	Tra Vinh Province	2022	10	Male	No	RS, GS, NS
13	PP498870	PP533561	CDV/VN-CTU/TV03	Tra Vinh Province	2022	24	Female	Yes	GS
14	PP498871	PP533562	CDV/VN-CTU/TV04	Tra Vinh Province	2022	6	Male	No	RS, NS
15	PP498872	PP533563	CDV/VN-CTU/TV05	Tra Vinh Province	2022	36	Female	No	RS
16	PP498873	PP533564	CDV/VN-CTU/TV06	Tra Vinh Province	2022	12	Female	No	RS, GS

17	PP498874	PP533565	CDV/VN-CTU/BT01	Ben Tre Province	2022	8	Female	No	RS
18	PP498875	PP533566	CDV/VN-CTU/BT02	Ben Tre Province	2022	24	Female	Yes	RS, GS
19	PP498876	PP533567	CDV/VN-CTU/BT03	Ben Tre Province	2022	84	Male	No	RS, GS
20	PP498877	PP533568	CDV/VN-CTU/BT04	Ben Tre Province	2023	11	Male	No	RS
21	PP498878	PP533569	CDV/VN-CTU/BT05	Ben Tre Province	2023	48	Male	No	GS
22	PP498879	PP533570	CDV/VN-CTU/CM01	Ca Mau Province	2022	36	Male	No	GS
23	PP498880	PP533571	CDV/VN-CTU/CM02	Ca Mau Province	2022	12	Female	Yes	RS, GS
24	PP498881	PP533572	CDV/VN-CTU/CM03	Ca Mau Province	2022	3	Male	No	RS, GS, NS
25	PP498882	PP533573	CDV/VN-CTU/CM04	Ca Mau Province	2023	48	Female	No	GS
26	PP498883	PP533574	CDV/VN-CTU/CM05	Ca Mau Province	2023	12	Female	Yes	RS, NS
27	PP498884	PP533575	CDV/VN-CTU/AG01	An Giang Province	2022	24	Female	No	RS, GS
28	PP498885	PP533576	CDV/VN-CTU/AG02	An Giang Province	2022	12	Female	Yes	RS, GS
29	PP498886	PP533577	CDV/VN-CTU/AG03	An Giang Province	2022	2	Male	No	RS, NS
30	PP498887	PP533578	CDV/VN-CTU/AG04	An Giang Province	2022	9	Male	No	RS
31	PP498888	PP533579	CDV/VN-CTU/AG05	An Giang Province	2022	7	Male	No	RS, GS
32	PP498889	PP533580	CDV/VN-CTU/AG06	An Giang Province	2022	12	Male	Yes	RS, GS
33	PP498890	PP533581	CDV/VN-CTU/AG07	An Giang Province	2022	48	Female	No	RS, GS
34	PP498891	PP533582	CDV/VN-CTU/AG08	An Giang Province	2022	72	Female	No	RS
35	PP498892	PP533583	CDV/VN-CTU/DT01	Dong Thap Province	2022	10	Male	No	GS

36	PP498893	PP533584	CDV/VN-CTU/DT02	Dong Thap Province	2022	24	Male	Yes	GS
37	PP498894	PP533585	CDV/VN-CTU/DT03	Dong Thap Province	2022	6	Male	Yes	RS, GS
38	PP498895	PP533586	CDV/VN-CTU/DT04	Dong Thap Province	2023	36	Female	No	RS, GS, NS
39	PP498896	PP533587	CDV/VN-CTU/DT05	Dong Thap Province	2023	12	Male	No	GS
40	PP498897	PP533588	CDV/VN-CTU/VL01	Vinh Long Province	2022	8	Female	Yes	RS, NS
41	PP498898	PP533589	CDV/VN-CTU/VL02	Vinh Long Province	2022	24	Female	No	RS, GS
42	PP498899	PP533590	CDV/VN-CTU/VL03	Vinh Long Province	2023	84	Female	No	RS, GS
43	PP498900	PP533591	CDV/VN-CTU/VL04	Vinh Long Province	2023	11	Female	No	RS, NS
44	PP498901	PP533592	CDV/VN-CTU/VL05	Vinh Long Province	2023	12	Male	No	RS
45	PP498902	PP533593	CDV/VN-CTU/VL06	Vinh Long Province	2023	2	Male	Yes	RS, GS, NS

RS: Respiratory sign (cough, dyspnea, purulent nasal discharge); GS: Gastrointestinal sign (vomiting, diarrhea); NS: Neurological sign (convulsion, myoclonus)
