# Supplementary material for: Epidemiological, Clinical, and Molecular Insights into Canine Distemper Virus in the Mekong Delta Region of Vietnam
Source: Viruses. 2025 May 29;17(6):781. doi: 10.3390/v17060781 (PMC12197358; doi:10.3390/v17060781)
Supplement: Supplementary file 1 [file viruses-17-00781-s001.zip › Table S2.pdf]

Table S2: Detailed information about reference CDV sequences based on GenBank database.

No.	H gene					F gene				
	GenBank accession number	Host	Genotype	Nation	Year	GenBank accession number	Host	Genotype	Nation	Year
1	FJ461698	Dog	Africa-1	South Africa	2007	KY971528	Wild dog	Africa-1	South Africa	2017
2	FJ461711	Dog	Africa-1	South Africa	2007	KY971532	Spotted hyena	Africa-1	South Africa	2017
3	FJ461713	Dog	Africa-1	South Africa	2007	KU578254	Jackal	Africa-2	Tanzania	2011
4	FJ461696	Dog	Africa-1	South Africa	2007	KU578257	Dog	Africa-2	Tanzania	1994
5	JN812976	Dog	Africa-2	Tanzania	1994	KU578256	Lion	Africa-2	Tanzania	1994
6	KC916714	Jackal	Africa-2	Tanzania	2011	AY964108	Dog	Arctic-like	USA	2004
7	KC916716	Fox	Africa-2	Tanzania	1994	AY964112	Dog	Arctic-like	USA	2005
8	AY964108	Dog	Arctic-like	USA	2004	KX024708	Badger	Arctic-like	Italy	2015
9	GQ214373	Dog	Arctic-like	Austria	2003	EF596901	Fox	Arctic-like	China	2007
10	DQ889178	Dog	Arctic-like	Hungary	2005	AB509345	Dog	Asia-1	Japan	2009
11	OM179846	Dog	Asia-1	Vietnam	2021	AB512286	Dog	Asia-1	Japan	2009
12	OM179847	Dog	Asia-1	Vietnam	2021	EU191985	Dog	Asia-1	Taiwan	2007
13	AB605890	Dog	Asia-1	Japan	2008	EU191986	Dog	Asia-1	Taiwan	2007
14	AB605891	Dog	Asia-1	Japan	2007	EU191987	Dog	Asia-1	Taiwan	2007

15	DQ887548	Dog	Asia-1	Taiwan	2005	EU191988	Dog	Asia-1	Taiwan	2007
16	EF445051	Dog	Asia-1	China	2007	EU191990	Dog	Asia-1	Taiwan	2007
17	EU325720	Dog	Asia-1	China	2007	EU191991	Dog	Asia-1	Taiwan	2007
18	EU716072	Dog	Asia-1	South Korea	2007	EU191992	Dog	Asia-1	Taiwan	2007
19	JX886776	Dog	Asia-1	Thailand	2010	EU191993	Dog	Asia-1	Taiwan	2007
20	JX886777	Dog	Asia-1	Thailand	2010	EU191995	Dog	Asia-1	Taiwan	2007
21	LC159583	Dog	Asia-1	Vietnam	2013	EU191996	Dog	Asia-1	Taiwan	2007
22	LC159584	Dog	Asia-1	Vietnam	2014	EU191997	Dog	Asia-1	Taiwan	2007
23	LC159586	Dog	Asia-1	Vietnam	2014	KP064129	Raccoon	Asia-1	China	2006
24	EU325726	Dog	Asia-1	China	2006	KX371581	Dog	Asia-1	China	2016
25	AB212965	Dog	Asia-1	Japan	2005	KX371582	Dog	Asia-1	China	2016
26	EU325730	Raccoon	Asia-1	China	2007	KX371583	Dog	Asia-1	China	2016
27	KU030831	Dog	Asia-1	China	2015	ON929297	Dog	Asia-1	South Korea	2019
28	KU521345	Mink	Asia-1	China	2014	ON929299	Dog	Asia-1	South Korea	2019
29	JQ732170	Raccoon	Asia-2	China	2005	KP769803	Panda	Asia-1	China	2014
30	FJ868166	Raccoon	Asia-2	South Korea	2009	EU192026	Dog	Asia-1	Taiwan	2007
31	AB212730	Dog	Asia-2	Japan	2005	AB753775	Dog	Asia-1	Japan	2012
32	AB025270	Dog	Asia-2	Japan	1999	AB753776	Dog	Asia-1	Japan	2012
33	EU252148	Dog	Asia-2	South Korea	2007	JN896331	Dog	Asia-1	China	2010

34	KJ437596	Dog	Asia-4	China	2012	EF596902	Raccoon	Asia-1	China	2007
35	KJ437594	Dog	Asia-4	China	2011	JN381189	Dog	Asia-1	China	2011
36	KJ489381	Dog	Asia-4	China	2012	KJ848781	Raccoon	Asia-1	China	2014
37	HM443718	Dog	Europe/South America-1	Italy	2010	EF596900	Raccoon	Asia-1	China	2007
38	Z77673	Dog	Europe/South America-1	Taiwan	1996	AB476402	Dog	Asia-2	Japan	2009
39	JN215473	Dog	Europe/South America-1	Uruguay	2007	AB476403	Dog	Asia-2	Japan	2009
40	DQ228166	Dog	Europe Wildlife	Italy	2005	AB474397	Dog	Asia-2	Japan	2008
41	JN153023	Raccoon	Europe Wildlife	Germany	2007	AB475100	Dog	Asia-2	Japan	2009
42	Z47759	Mink	Europe Wildlife	Denmark	1995	AB475099	Dog	Asia-2	Japan	2009
43	AY548109	Raccoon	North America-1	USA	1996	HM063009	Mink	Asia-3	Kazakhstan	1989
44	AY542312	Raccoon	North America-1	USA	1996	HM046486	Seal	Asia-3	Kazakhstan	2007
45	AF112189	Dog	North America-2	USA	1989	MF437053	Dog	Europe/South America-1	Gabon	2015
46	Z47764	Javelina	North America-2	USA	1989	AY386315	Ferret	Europe/South America-1	USA	2003
47	AY465925	Raccoon	North America-2	USA	2001	AY386316	Ferret	Europe/South America-1	USA	2003
48	KT266738	Dog	North America-3	Mexico	2013	KM280689	Dog	Europe/South America-1	Uruguay	2012
49	JN836737	Martes pennanti	North America-3	USA	2009	AY445077	Raccoon	North America-1	USA	1998
50	FJ392651	Dog	South America-2	Argentina	2005	AY542312	Raccoon	North America-1	USA	1998
51	KF835422	Dog	South America-3	Colombia	2012	AY466011	Raccoon	North America-1	USA	1998
52	KF835411	Dog	South America-3	Colombia	2011	AY649446	Raccoon	North America-2	USA	2004

53	KJ747371	Dog	South/North America-4	USA	2013	EU716337	Dog	North America-2	USA	2004
54	KJ747372	Dog	South/North America-4	USA	2013	AY964110	Dog	North America-3	USA	2005
55	MN702774	Dog	Rockborn-like	India	2019	KT224718	Dog	South America-2	Argentina	2005
56	GU810819	Dog	Rockborn-like	Sweden	2010	KT224719	Dog	South America-2	Argentina	2010
57	AF259552		Vaccine/Snyder Hill	Russia	2000	KT224732	Dog	South America-2	Argentina	2014
58	Z35493		Vaccine/Convac	Denmark	1994	MT012802	Dog	South America-3	Colombia	2012
59	FJ461701		Vaccine/Nobivac DHPPI	South Africa	2008	MT012803	Dog	South/North America-4	Colombia	2017
60	FJ461708		Vaccine/Galaxy DA2PPV	South Africa	2008	KJ747371	Fox	South/North America-4	USA	2013
61	FJ461709		Vaccine/Nobivac PuppyDP	South Africa	2008	KJ747372	Dog	South/North America-4	USA	2013
62	FJ461710		Vaccine/Canigen DHPPI	South Africa	2008	AY964114	Dog	Rockborn-like	USA	2004
63	AF378705		Vaccine/Onderstepoort	USA	2001	AF378705		Vaccine/Onderstepoort	USA	2001
64	EU143737		Vaccine/Onderstepoort	USA	2007	NC001921		Vaccine/Onderstepoort	USA	1997
65	NC001921		Vaccine/Onderstepoort	USA	1997	GU138403		Vaccine/Snyder Hill	Canada	2009
