# Supplementary material for: Epidemiological, Clinical, and Molecular Insights into Canine Distemper Virus in the Mekong Delta Region of Vietnam
Source: Viruses. 2025 May 29;17(6):781. doi: 10.3390/v17060781 (PMC12197358; doi:10.3390/v17060781)
Supplement: Supplementary file 1 [file viruses-17-00781-s001.zip › Table S3.pdf]

Table S3: Potential N-linked glycosylation sites of H gene of CDV sequences collected from dogs raised in MD region of Vietnam.

No.	Sequence code	Position	Potential	Jury agreement	N-glycosylation result
1	PP498858	391 NQTS	0.7223	(9/9)	++
		422 NISF	0.6160	(8/9)	+
		456 NGTV	0.4400	(6/9)	-
2	PP498859	391 NQTS	0.7263	(9/9)	++
		422 NISF	0.6160	(8/9)	+
		456 NGTV	0.4400	(6/9)	-
3	PP498860	391 NQTS	0.7263	(9/9)	++
		422 NISF	0.6160	(8/9)	+
		456 NGTV	0.4400	(6/9)	-
4	PP498861	391 NQTS	0.7262	(9/9)	++
		422 NISF	0.6160	(8/9)	+
		456 NGTV	0.4400	(6/9)	-
5	PP498862	391 NQTS	0.7263	(9/9)	++
		422 NISF	0.6159	(8/9)	+
		456 NGTV	0.4400	(6/9)	-
6	PP498863	391 NQTS	0.7262	(9/9)	++
		422 NISF	0.6158	(8/9)	+
		456 NGTV	0.4400	(6/9)	-
7	PP498864	391 NQTS	0.7263	(9/9)	++
		422 NISF	0.6159	(8/9)	+
		456 NGTV	0.4400	(6/9)	-
8	PP498865	391 NQTS	0.7263	(9/9)	++
		422 NISF	0.6159	(8/9)	+
		456 NGTV	0.4400	(6/9)	-
9	PP498866	391 NQTS	0.7263	(9/9)	++
		422 NISF	0.6159	(8/9)	+
		456 NGTV	0.4400	(6/9)	-

10	PP498867	391 NQTS	0.7263	(9/9)	++
		422 NISF	0.6159	(8/9)	+
		456 NGTV	0.4400	(6/9)	-
11	PP498868	391 NQTS	0.7264	(9/9)	++
		422 NISF	0.5988	(7/9)	+
		456 NGTV	0.4367	(6/9)	-
12	PP498869	391 NQTS	0.7260	(9/9)	++
		422 NISF	0.6159	(8/9)	+
		456 NGTV	0.4400	(6/9)	-
13	PP498870	391 NQTS	0.7263	(9/9)	++
		422 NISF	0.5989	(7/9)	+
		456 NGTV	0.4401	(6/9)	-
14	PP498871	391 NQTS	0.7263	(9/9)	++
		422 NISF	0.5989	(8/9)	+
		456 NGTV	0.4400	(6/9)	-
15	PP498872	391 NQTS	0.7268	(9/9)	++
		422 NISF	0.6160	(8/9)	+
		456 NGTV	0.4400	(6/9)	-
16	PP498873	391 NQTS	0.7262	(9/9)	++
		422 NISF	0.6159	(8/9)	+
		456 NGTV	0.4399	(6/9)	-
17	PP498874	391 NQTS	0.7217	(9/9)	++
		422 NISF	0.6428	(9/9)	++
		456 NGTV	0.4109	(6/9)	-
18	PP498875	391 NQTS	0.7217	(9/9)	++
		422 NISF	0.6428	(9/9)	++
		456 NGTV	0.4109	(6/9)	-
19	PP498876	391 NQTS	0.7262	(9/9)	++
		422 NISF	0.6158	(8/9)	+
		456 NGTV	0.4400	(6/9)	-

20	PP498877	391 NQTS	0.7262	(9/9)	++
		422 NISF	0.6160	(8/9)	+
		456 NGTV	0.4400	(6/9)	-
21	PP498878	391 NQTS	0.7254	(9/9)	++
		422 NISF	0.6168	(8/9)	+
		456 NGTV	0.4409	(6/9)	-
22	PP498879	391 NQTS	0.7266	(9/9)	++
		422 NISF	0.6162	(8/9)	+
		456 NGTV	0.4404	(6/9)	-
23	PP498880	391 NQTS	0.7264	(9/9)	++
		422 NISF	0.6159	(8/9)	+
		456 NGTV	0.4400	(6/9)	-
24	PP498881	391 NQTS	0.7217	(9/9)	++
		422 NISF	0.6428	(9/9)	++
		456 NGTV	0.4109	(6/9)	-
25	PP498882	391 NQTS	0.7263	(9/9)	++
		422 NISF	0.5988	(7/9)	+
		456 NGTV	0.4368	(6/9)	-
26	PP498883	391 NQTS	0.7264	(9/9)	++
		422 NISF	0.5988	(7/9)	+
		456 NGTV	0.4367	(6/9)	-
27	PP498884	391 NQTS	0.7264	(9/9)	++
		422 NISF	0.6159	(8/9)	+
		456 NGTV	0.4400	(6/9)	-
28	PP498885	391 NQTS	0.7263	(9/9)	++
		422 NISF	0.6159	(8/9)	+
		456 NGTV	0.4400	(6/9)	-
29	PP498886	391 NQTS	0.7263	(9/9)	++
		422 NISF	0.6159	(8/9)	+
		456 NGTV	0.4400	(6/9)	-

30	PP498887	391 NQTS	0.7263	(9/9)	++
		422 NISF	0.6159	(8/9)	+
		456 NGTV	0.4400	(6/9)	-
31	PP498888	391 NQTS	0.7263	(9/9)	++
		422 NISF	0.6159	(8/9)	+
		456 NGTV	0.4400	(6/9)	-
32	PP498889	391 NQTS	0.7264	(9/9)	++
		422 NISF	0.6159	(8/9)	+
		456 NGTV	0.4400	(6/9)	-
33	PP498890	391 NQTS	0.7217	(9/9)	++
		422 NISF	0.6428	(9/9)	++
		456 NGTV	0.4109	(6/9)	-
34	PP498891	391 NQTS	0.7261	(9/9)	++
		422 NISF	0.6160	(8/9)	+
		456 NGTV	0.4399	(6/9)	-
35	PP498892	391 NQTS	0.6831	(9/9)	++
		422 NISF	0.6160	(8/9)	+
		456 NGTV	0.4400	(6/9)	-
36	PP498893	391 NQTS	0.6831	(9/9)	++
		422 NISF	0.6160	(8/9)	+
		456 NGTV	0.4400	(6/9)	-
37	PP498894	391 NQTS	0.7263	(9/9)	++
		422 NISF	0.6159	(8/9)	+
		456 NGTV	0.4400	(6/9)	-
38	PP498895	391 NQTS	0.7261	(9/9)	++
		422 NISF	0.6160	(8/9)	+
		456 NGTV	0.4399	(6/9)	-
39	PP498896	391 NQTS	0.7264	(9/9)	++
		422 NISF	0.6159	(8/9)	+
		456 NGTV	0.4400	(6/9)	-

40	PP498897	391 NQTS	0.7266	(9/9)	++
		422 NISF	0.6162	(8/9)	+
		456 NGTV	0.4404	(6/9)	-
41	PP498898	391 NQTS	0.7266	(9/9)	++
		422 NISF	0.6162	(8/9)	+
		456 NGTV	0.4404	(6/9)	-
42	PP498899	391 NQTS	0.7264	(9/9)	++
		422 NISF	0.6159	(8/9)	+
		456 NGTV	0.4400	(6/9)	-
43	PP498900	391 NQTS	0.7264	(9/9)	++
		422 NISF	0.6159	(8/9)	+
		456 NGTV	0.4400	(6/9)	-
44	PP498901	391 NQTS	0.7263	(9/9)	++
		422 NISF	0.6159	(8/9)	+
		456 NGTV	0.4400	(6/9)	-
45	PP498902	391 NQTS	0.7263	(9/9)	++
		422 NISF	0.6159	(8/9)	+
		456 NGTV	0.4400	(6/9)	-
