# Supplementary material for: Epidemiological, Clinical, and Molecular Insights into Canine Distemper Virus in the Mekong Delta Region of Vietnam
Source: Viruses. 2025 May 29;17(6):781. doi: 10.3390/v17060781 (PMC12197358; doi:10.3390/v17060781)
Supplement: Supplementary file 1 [file viruses-17-00781-s001.zip › Table S4.pdf]

Table S4: Potential N-linked glycosylation sites of F gene of CDV sequences collected from dogs raised in MD region of Vietnam.

No.	Sequence code	Position	Potential	Jury agreement	N-glycosylation result
1	PP533549	62 NGTR	0.6698	(9/9)	++
		108 NATN	0.5459	(5/9)	+
		141 NLST	0.4984	(4/9)	-
		173 NVSL	0.6930	(9/9)	++
		179 NCTK	0.6384	(7/9)	+
2	PP533550	62 NRTR	0.6714	(9/9)	++
		108 NATN	0.5459	(5/9)	+
		141 NLST	0.4983	(4/9)	-
		173 NVSL	0.6892	(9/9)	++
		179 NCTK	0.6749	(8/9)	+
3	PP533551	62 NGTR	0.7130	(9/9)	++
		108 NATN	0.5459	(5/9)	+
		141 NLST	0.4983	(4/9)	-
		173 NVSL	0.6890	(9/9)	++
		179 NCTK	0.6747	(8/9)	+
4	PP533552	62 NRTR	0.6497	(9/9)	++
		108 NATN	0.5460	(5/9)	+
		141 NLST	0.4982	(4/9)	-
		173 NVSL	0.6920	(9/9)	++
		179 NCTK	0.6961	(9/9)	++
5	PP533553	62 NRTR	0.6716	(9/9)	++
		108 NATN	0.5459	(5/9)	+
		141 NLST	0.4984	(4/9)	-
		173 NVSL	0.6891	(9/9)	++
		179 NCTK	0.6747	(8/9)	+
6	PP533554	62 NGTR	0.6699	(9/9)	++
		108 NATN	0.5460	(5/9)	+

		141 NLST	0.4984	(4/9)	-
		173 NVSL	0.6921	(9/9)	++
		179 NCTK	0.6960	(9/9)	++
		62 NRTR	0.6851	(9/9)	++
		108 NATN	0.5460	(5/9)	+
7	PP533555	141 NLST	0.4986	(4/9)	-
		173 NVSL	0.6891	(9/9)	++
		179 NCTK	0.6749	(8/9)	+
		62 NRTR	0.6782	(9/9)	++
		108 NATN	0.5460	(5/9)	+
8	PP533556	141 NLST	0.4983	(4/9)	-
		173 NVSL	0.6890	(9/9)	++
		179 NCTK	0.6749	(8/9)	+
		62 NRTR	0.6714	(9/9)	++
		108 NATN	0.5460	(5/9)	+
9	PP533557	141 NLST	0.4984	(4/9)	-
		173 NVSL	0.6890	(9/9)	++
		179 NCTK	0.6748	(8/9)	+
		62 NGTR	0.6814	(9/9)	++
		108 NATN	0.5458	(5/9)	+
10	PP533558	141 NLST	0.4981	(4/9)	-
		173 NVSL	0.6919	(9/9)	++
		179 NCTK	0.6960	(9/9)	++
		62 NRTR	0.6782	(9/9)	++
		108 NATN	0.5459	(5/9)	+
11	PP533559	141 NLST	0.4984	(4/9)	-
		173 NVSL	0.6891	(9/9)	++
		179 NCTK	0.6748	(8/9)	+
12	PP533560	62 NRTR	0.6849	(9/9)	++
		108 NATN	0.5280	(5/9)	+

		141 NLST	0.4981	(4/9)	-
		173 NVSL	0.6922	(9/9)	++
		179 NCTK	0.7089	(9/9)	++
13	PP533561	62 NRTR	0.6776	(9/9)	++
		108 NATN	0.5278	(5/9)	+
		141 NLST	0.4980	(4/9)	-
		173 NVSL	0.6889	(9/9)	++
		179 NCTK	0.6747	(8/9)	+
14	PP533562	62 NRTR	0.6849	(9/9)	++
		108 NATN	0.5279	(5/9)	+
		141 NLST	0.4980	(4/9)	-
		173 NVSL	0.6889	(9/9)	++
		179 NCTK	0.6749	(8/9)	+
15	PP533563	62 NRTR	0.6781	(9/9)	++
		108 NATN	0.5041	(5/9)	+
		141 NLST	0.4980	(4/9)	-
		173 NVSL	0.6821	(9/9)	++
		179 NCTK	0.6748	(8/9)	+
16	PP533564	62 NRTR	0.6781	(9/9)	++
		108 NATN	0.5040	(5/9)	+
		141 NLST	0.4979	(4/9)	-
		173 NVSL	0.6821	(9/9)	++
		179 NCTK	0.6747	(8/9)	+
17	PP533565	62 NGTR	0.6764	(9/9)	++
		108 NATN	0.5461	(5/9)	+
		141 NLST	0.4983	(4/9)	-
		173 NVSL	0.6923	(9/9)	++
		179 NCTK	0.7089	(9/9)	++
18	PP533566	62 NRTR	0.6782	(9/9)	++
		108 NATN	0.5400	(5/9)	+

		141 NLST	0.4982	(4/9)	-
		173 NVSL	0.6889	(9/9)	++
		179 NCTK	0.6748	(8/9)	+
19	PP533567	62 NRTR	0.6782	(9/9)	++
		108 NATN	0.5460	(5/9)	+
		141 NLST	0.4986	(4/9)	-
		173 NVSL	0.6891	(9/9)	++
		179 NCTK	0.6748	(8/9)	+
20	PP533568	62 NRTR	0.6782	(9/9)	++
		108 NATN	0.5400	(5/9)	+
		141 NLST	0.4982	(4/9)	-
		173 NVSL	0.6868	(9/9)	++
		179 NCTK	0.6843	(9/9)	++
21	PP533569	62 NRTR	0.6782	(9/9)	++
		108 NATN	0.5400	(5/9)	+
		141 NLST	0.4982	(4/9)	-
		173 NVSL	0.6889	(9/9)	++
		179 NCTK	0.6748	(8/9)	+
22	PP533570	62 NRTR	0.6781	(9/9)	++
		108 NATN	0.5400	(5/9)	+
		141 NLST	0.4979	(4/9)	-
		173 NVSL	0.6821	(9/9)	++
		179 NCTK	0.6747	(8/9)	+
23	PP533571	62 NRTR	0.6782	(9/9)	++
		108 NATN	0.5400	(5/9)	+
		141 NLST	0.4982	(4/9)	-
		173 NVSL	0.6889	(9/9)	++
		179 NCTK	0.6748	(8/9)	+
24	PP533572	62 NRTR	0.6782	(9/9)	++
		108 NATN	0.5400	(5/9)	+

		141 NLST	0.4982	(4/9)	-
		173 NVSL	0.6889	(9/9)	++
		179 NCTK	0.6748	(8/9)	+
25	PP533573	62 NRTR	0.6782	(9/9)	++
		108 NATN	0.5400	(5/9)	+
		141 NLST	0.4982	(4/9)	-
		173 NVSL	0.6889	(9/9)	++
		179 NCTK	0.6748	(8/9)	+
26	PP533574	62 NGTR	0.7066	(9/9)	++
		108 NATN	0.5459	(5/9)	+
		141 NLST	0.4983	(4/9)	-
		173 NVSL	0.6923	(9/9)	++
		179 NCTK	0.7089	(9/9)	++
27	PP533575	62 NRTR	0.6781	(9/9)	++
		108 NATN	0.5041	(5/9)	+
		141 NLST	0.4980	(4/9)	-
		173 NVSL	0.6821	(9/9)	++
		179 NCTK	0.6748	(8/9)	+
28	PP533576	62 NRTR	0.6781	(9/9)	++
		108 NATN	0.5040	(5/9)	+
		141 NLST	0.4979	(4/9)	-
		173 NVSL	0.6821	(9/9)	++
		179 NCTK	0.6747	(8/9)	+
29	PP533577	62 NGTR	0.6763	(9/9)	++
		108 NATN	0.5252	(5/9)	+
		141 NLST	0.4980	(4/9)	-
		173 NVSL	0.6820	(9/9)	++
		179 NCTK	0.6747	(8/9)	+
30	PP533578	62 NRTR	0.6781	(9/9)	++
		108 NATN	0.5040	(5/9)	+

		141 NLST	0.4979	(4/9)	-
		173 NVSL	0.6821	(9/9)	++
		179 NCTK	0.6747	(8/9)	+
31	PP533579	62 NRTR	0.6781	(9/9)	++
		108 NATN	0.5040	(5/9)	+
		141 NLST	0.4979	(4/9)	-
		173 NVSL	0.6821	(9/9)	++
		179 NCTK	0.6747	(8/9)	+
32	PP533580	62 NRTR	0.6779	(9/9)	++
		108 NATN	0.5062	(5/9)	+
		141 NLST	0.4978	(4/9)	-
		173 NVSL	0.6820	(9/9)	++
		179 NCTK	0.6746	(8/9)	+
33	PP533581	62 NGTR	0.7062	(9/9)	++
		108 NATN	0.5194	(5/9)	+
		141 NLST	0.4980	(4/9)	-
		173 NVSL	0.6821	(9/9)	++
		179 NCTK	0.6747	(8/9)	+
34	PP533582	62 NRTR	0.6776	(9/9)	++
		108 NATN	0.5058	(5/9)	+
		141 NLST	0.4977	(4/9)	-
		173 NVSL	0.6889	(9/9)	++
		179 NCTK	0.6742	(8/9)	+
35	PP533583	62 NGTR	0.7066	(9/9)	++
		108 NATN	0.5459	(5/9)	+
		141 NLST	0.4983	(4/9)	-
		173 NVSL	0.6923	(9/9)	++
		179 NCTK	0.7089	(9/9)	++
36	PP533584	62 NRTR	0.6793	(9/9)	++
		108 NATN	0.5459	(5/9)	+

		141 NLST	0.4984	(4/9)	-
		173 NVSL	0.6891	(9/9)	++
		179 NCTK	0.6749	(8/9)	+
37	PP533585	62 NRTR	0.6782	(9/9)	++
		108 NATN	0.5460	(5/9)	+
		141 NLST	0.4986	(4/9)	-
		173 NVSL	0.6891	(9/9)	++
		179 NCTK	0.6748	(8/9)	+
38	PP533586	62 NGTR	0.7066	(9/9)	++
		108 NATN	0.5459	(5/9)	+
		141 NLST	0.4983	(4/9)	-
		173 NVSL	0.6923	(9/9)	++
		179 NCTK	0.7089	(9/9)	++
39	PP533587	62 NGTR	0.6764	(9/9)	++
		108 NATN	0.5461	(5/9)	+
		141 NLST	0.4983	(4/9)	-
		173 NVSL	0.6923	(9/9)	++
		179 NCTK	0.7089	(9/9)	++
40	PP533588	62 NGTR	0.6764	(9/9)	++
		108 NATN	0.5461	(5/9)	+
		141 NLST	0.4983	(4/9)	-
		173 NVSL	0.6923	(9/9)	++
		179 NCTK	0.7089	(9/9)	++
41	PP533589	62 NRTR	0.6782	(9/9)	++
		108 NATN	0.5460	(5/9)	+
		141 NLST	0.4986	(4/9)	-
		173 NVSL	0.6891	(9/9)	++
		179 NCTK	0.6748	(8/9)	+
42	PP533590	62 NGTR	0.7066	(9/9)	++
		108 NATN	0.5459	(5/9)	+

		141 NLST	0.4983	(4/9)	-
		173 NVSL	0.6923	(9/9)	++
		179 NCTK	0.7089	(9/9)	++
		62 NRTR	0.6780	(9/9)	++
		108 NATN	0.5197	(5/9)	+
43	PP533591	141 NLST	0.4982	(4/9)	-
		173 NVSL	0.6923	(9/9)	++
		179 NCTK	0.7089	(9/9)	++
		62 NRTR	0.6778	(9/9)	++
		108 NATN	0.5282	(5/9)	+
44	PP533592	141 NLST	0.4982	(4/9)	-
		173 NVSL	0.6889	(9/9)	++
		179 NCTK	0.6748	(8/9)	+
		62 NRTR	0.6778	(9/9)	++
		108 NATN	0.5282	(5/9)	+
45	PP533593	141 NLST	0.4982	(4/9)	-
		173 NVSL	0.6889	(9/9)	++
		179 NCTK	0.6748	(8/9)	+
